# A Modified Suture Hook Technique for Lateral Meniscus Tears: Chinese Union Suture Procedure (CUSP)

**DOI:** 10.1016/j.eats.2024.102946

**Published:** 2024-04-10

**Authors:** Wenbo Yang, Yi Li, Shuyan Wu, Wei Yu, Chunqing Meng, Hong Wang, Wei Huang

**Affiliations:** Department of Orthopaedics, Union Hospital, Tongji Medical College, Huazhong University of Science and Technology, Wuhan, China

## Abstract

Tears in the lateral meniscus, especially the body and posterior horn, are a common sports medicine injury, and severe cases require arthroscopic treatment. However, the narrow space in the posterolateral compartment of the knee joint makes the surgeon‘s operation inconvenient. Here we have proposed a suture technique suitable for lateral meniscus posterior horn/body injuries known as the Chinese Union Suture Procedure. The introduction of the posteromedial-transseptal portal allows for a suitable angle of operation for suture hooks in case of injuries to the posterior horn of the lateral meniscus or the popliteal hiatus area of the lateral meniscus. The use of the continuous sewing machine–like suture technique allows surgeons to more quickly and flexibly address meniscus tears. We have also introduced a retractor as an auxiliary tool for portal establishment. In conclusion, our improved technique enables vertical mattress suturing for meniscus tears, and it allows for tying knots on the tibial surface of the meniscus, which is challenging to achieve with traditional all-inside suture hook techniques. Our technique combines flexibility, speed, and cost-effectiveness, making it valuable for clinical applications.

The locations of meniscal tears are diverse and can involve tears in the anterior horn, body, posterior horn, and root of the meniscus. The types of meniscal tears include longitudinal tears, bucket-handle tears, and radial tears. Due to the complex locations and types of meniscal tears, various suturing techniques are employed in clinical practice, such as the outside-in technique, inside-out technique, all-inside suture hook technique, and all-inside suture device technique.^1^ However, these suturing techniques each have their limitations. Based on the aforementioned clinical situation, we previously reported an improved continuous suturing method using a suture hook for all-inside suturing, which is suitable for various tear types. This method is known as the continuous sewing machine–like suture technique.^2^ The improved suturing technique primarily involves adding an auxiliary portal and using a single suture for multiple continuous threading and suture. This technique offers greater flexibility in selecting the suture and knotting locations. Continuous suturing can also enhance the strength of the meniscus and is more clinically applicable. However, it should be noted that, despite our improved technique being suitable for different types of injuries in various locations of both the medial and lateral meniscus, some tear sites still pose challenges during surgery. For example, tears in the posterior horn of the lateral meniscus and in the popliteal hiatus are currently recognized as challenging types to manage. One significant reason for the difficulties encountered is the presence of important structures such as the genicular nerves and the popliteal tendon in the posterior lateral aspect of the knee joint. Traditional anteromedial or anterolateral portals pose greater exposure and operational challenges when dealing with the lateral meniscus especially the popliteomeniscal fascicles and posterior horn. To further address the issue of limited surgical access for posterior horn injuries of the lateral meniscus, we previously reported an improved posteromedial portal, known as the posteromedial-transseptal portal, for suturing the lateral meniscus especially the popliteomeniscal fascicles and posterior horn.^3^ Through this improved technique, the suture hook can be operated with better visibility, and the operation positions become more flexible. Repairing tears in the the lateral meniscus will be easier, quicker, and without the occurrence of vascular, tendon, or nerve complications using this improved technique.

However, this improved posteromedial-transseptal portal also carries a certain risk of vascular and nerve injury during its creation process, and the suture hook cannot achieve a vertical mattress all-inside suture. To handle various types of lateral meniscus posterior horn/body tears more simply and quickly, we have combined and further improved 2 previously reported techniques, resulting in the Chinese Union Suture Procedure (CUSP). This technique has a broad application scope, offering technical advantages, especially for lateral meniscus injuries in the popliteal hiatus area, posterior horn, and posterior root, particularly for tears close to the synovial edge. Our improved technique also does not require the use of expensive excess consumables, making it more economically valuable and applicability.

## Surgical Technique

**Fig 1:**
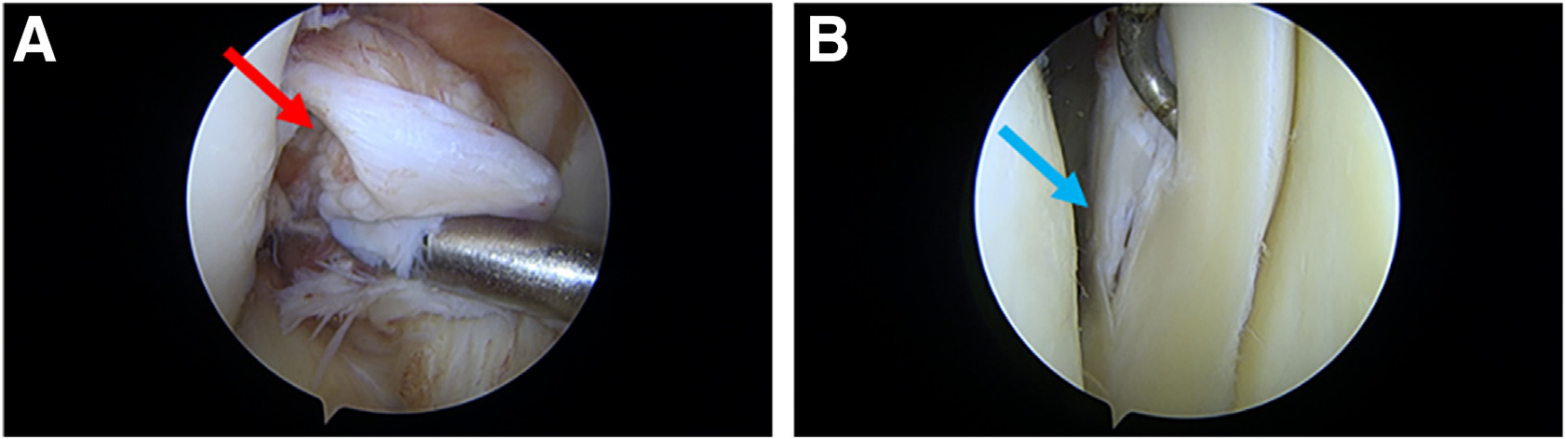
Conventional anteromedial and anterolateral portals are established to observe the exposed lesions. In this case, the patient had a concurrent anterior cruciate ligament injury and a longitudinal tear in the posterior horn of the lateral meniscus (right knee). The red arrow in A indicates the rupture of the anterior cruciate ligament, while the blue arrow in B points to the longitudinal tear in the posterior horn of the lateral meniscus.

A brief introduction to our surgical techniques is presented in Video 1. First, conventional anteromedial and anterolateral portals are established to observe the exposed lesions (Fig 1). The core content of our improved technology consists of 3 main steps.

### Step 1: Establish the Posteromedial Portal

**Fig 2:**
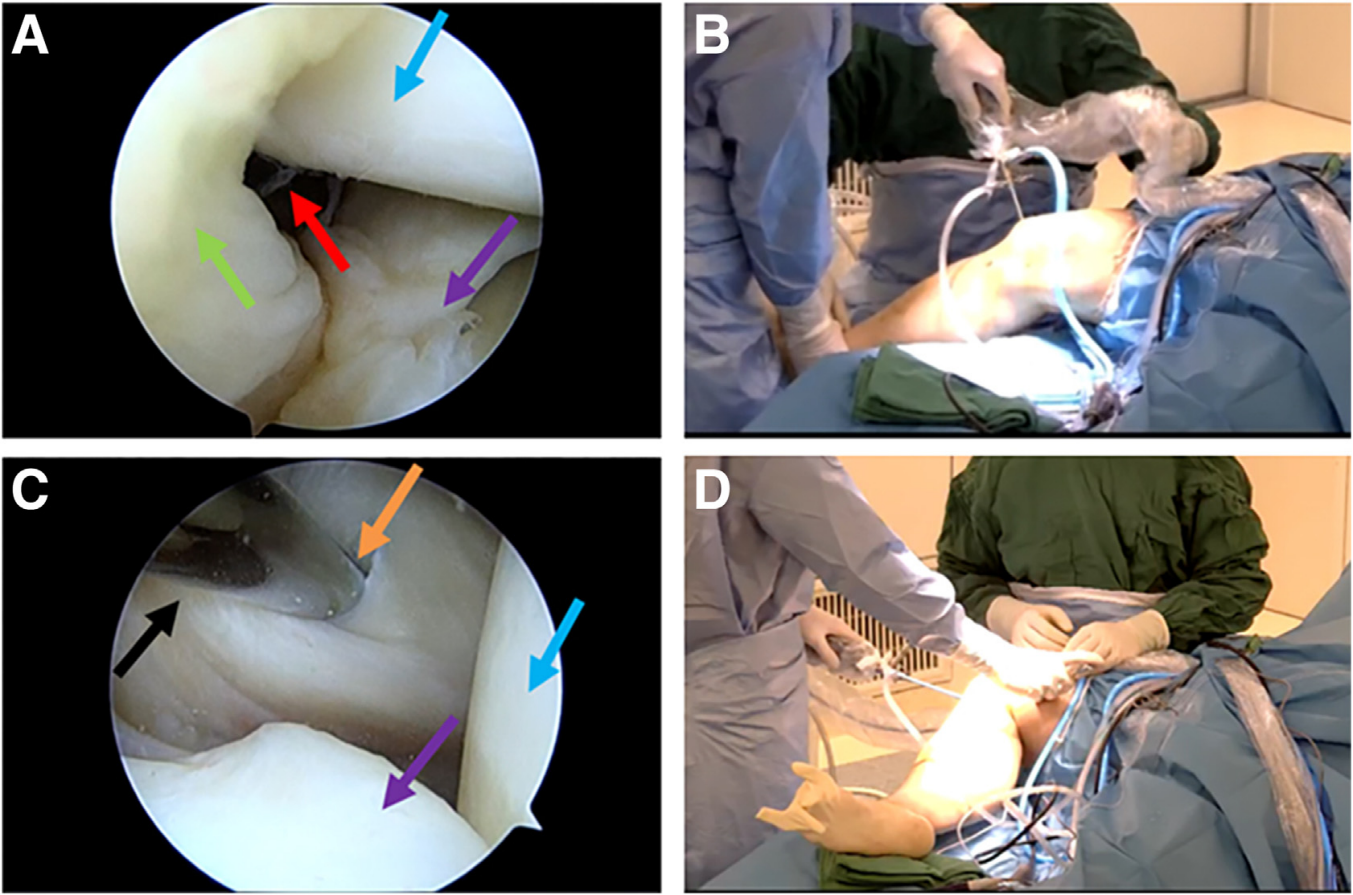
The posteromedial portal is established and blunt trocar obturator enters the posteromedial compartment of the right knee joint. (A, B) With the knee joint flexed at 15 to 30 degrees, the anterolateral portal is used as the observation portal. The arthroscopic lens passes through the gap between the posterior cruciate ligament and the medial condyle of the femur to enter the posteromedial compartment. (A) Intra-articular view. (B) Extra-articular view. (C, D) With the knee joint in the “figure-4” position, using the anterolateral portal as the observation portal and establishing the standard posteromedial portal, the blunt trocar obturator is inserted into the posteromedial compartment. (C) Intra-articular view. (D) Extra-articular view. The green arrow indicates the posterior cruciate ligament, the blue arrow indicates the medial condyle of the femur, the red arrow indicates the posteromedial compartment, the purple arrow indicates the posterior horn of the medial meniscus, the orange arrow indicates the posteromedial portal, and the black arrow indicates the blunt trocar obturator.

**Fig 3:**
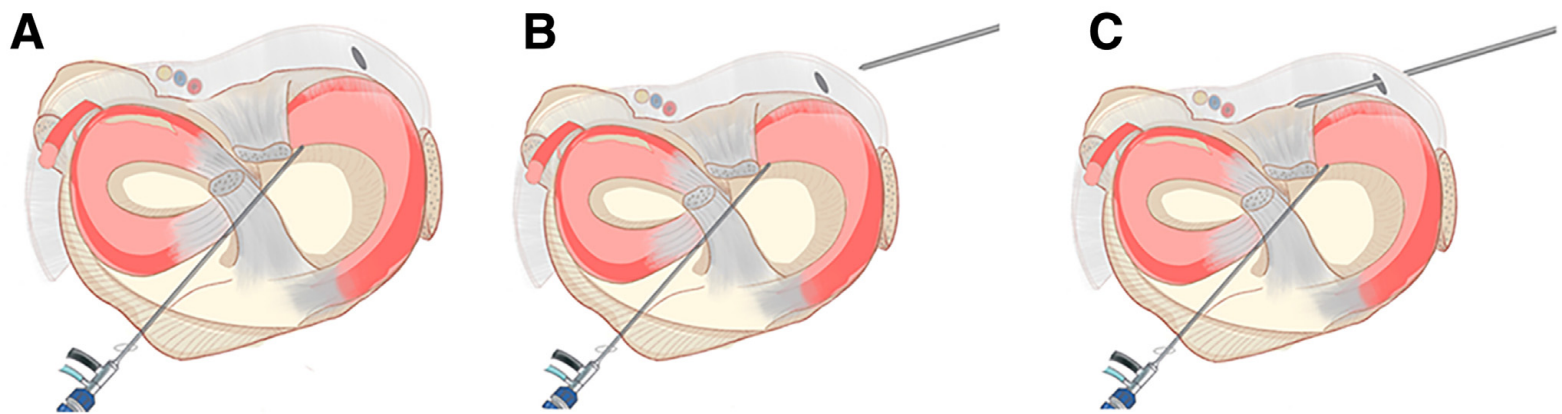
The schematic diagram of establishing the posteromedial portal. (A-C) Step-by-step process of inserting the arthroscopic lens through the gap between the posterior cruciate ligament and the medial condyle of the femur to enter into the posteromedial compartment, establishing the posteromedial operation portal, and inserting the blunt trocar obturator into the posteromedial compartment through the posteromedial portal.

**Fig 4:**
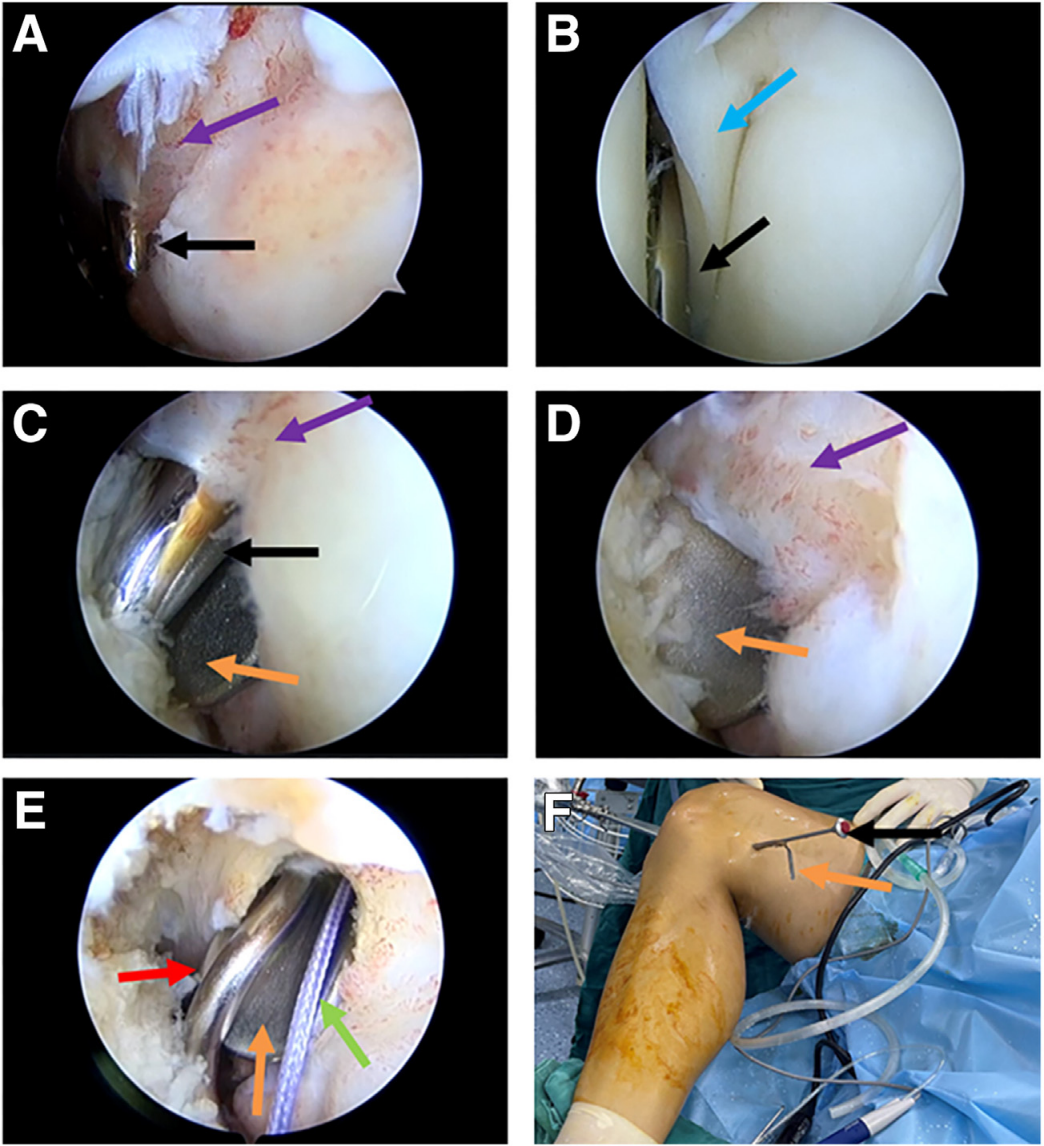
The process of establishing the posteromedial-transseptal portal. (A, B) To position the right knee joint in the “figure-4 sign,” the blunt trocar obturator is passed bluntly from behind the posterior cruciate ligament to the posterolateral compartment through the posteromedial portal. The arthroscopic lens remains in the anterolateral portal, but the field of view transitions from the posteromedial compartment to the posterolateral compartment. At this point, the blunt trocar obturator precisely reaches the location of the tear in the posterior horn of the lateral meniscus. (C-E) The process is demonstrated step by step, showing the insertion of the retractor along the blunt trocar obturator, followed by the removal of the blunt trocar obturator. Then, the suture hook is inserted along the established posteromedial-transseptal portal. (F) Extra view shows the process of establishing the posteromedial-transseptal portal. The black arrow indicates the blunt trocar obturator, the purple arrow indicates the posterior mediastinum, the blue arrow indicates the posterior horn of the lateral meniscus, the orange arrow indicates the retractor, the red arrow indicates the suture hook, and the green arrow indicates the Orthocord suture loaded within the suture hook.

The surgeon first positions the knee joint at 15 to 30 degrees of flexion and uses the anterolateral portal as the observation portal, inserting the arthroscope into the posteromedial compartment to prepare for guiding the establishment of the posteromedial portal (Fig 2 A and B). Specifically, the arthroscope is inserted into the posteromedial compartment through the gap between the posterior cruciate ligament, the medial condyle of the femur, and the posterior root of the medial meniscus. Then, the knee joint is positioned in the “figure-4 sign,“ establishing a standard posteromedial portal, and the blunt trocar obturator is inserted through the posteromedial portal into the posteromedial compartment (Fig 2 C and D). Figure 3 shows the schematic diagram of establishing the posteromedial portal.

### Step 2: Establish the Posteromedial-Transseptal Portal for Lateral Meniscus Repair

**Fig 5:**
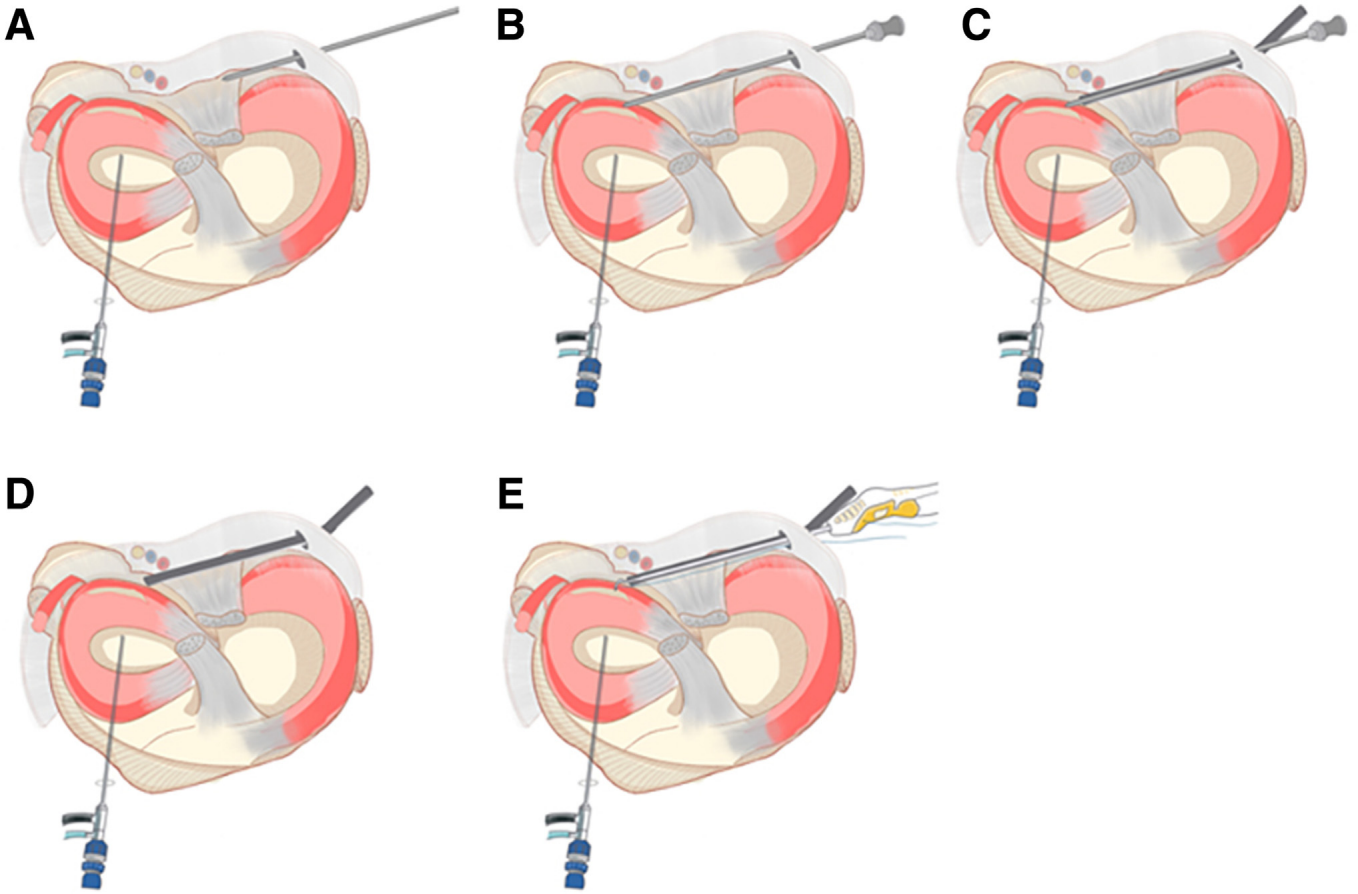
Illustration of the complete process of establishing a posteromedial-transseptal portal. (A) The arthroscopic lens remains in the anterolateral portal, but the field of view transitions from the posteromedial compartment to the posterolateral compartment. (B) The blunt trocar obturator is inserted through the posteromedial portal, passing through the gap between the posterior cruciate ligament and the joint capsule, into the posterolateral compartment, reaching the tear area of the lateral meniscus. (C) Insertion of the retractor along the blunt trocar obturator. (D) Removal of the blunt trocar obturator. (E) Insertion of the suture hook loaded with sutures.

The patient’s knee joint is still in the “figure-4 sign.” Under arthroscopic monitoring, the blunt trocar obturator, which has been inserted into the posteromedial compartment, is passed from the gap between the posterior cruciate ligament and the posterior joint capsule of the knee joint into the posterolateral compartment. During this process, the arthroscopic view transitions from the posteromedial compartment to the posterolateral compartment. The arthroscope remains in the anterolateral portal. At this point, the blunt trocar obturator has reached the location of the damaged posterior horn of the lateral meniscus (Fig 4 A and B). A retractor (Mitek Surgical Malleable Graft Retractor with Meniscal Deployment Gun; Depuy Mitek) for soft tissue isolation (Fig 4C) is placed along the blunt trocar obturator and the blunt trocar obturator is removed (Fig 4D). The complete posteromedial-transseptal portal for operation is set up. Due to the use of a retractor for soft tissue isolation, the suture hook loaded with sutures can easily glide along the portal into the posterolateral compartment of the knee joint, without worrying about vascular and nerve injury. The surgeon can hold the arthroscope and suture hook in hand to perform the subsequent surgical procedures (Fig 4E). Figure 4F provides an external view of this process. Figure 5 illustrates this step schematically.

### Step 3: Repair the Lateral Meniscus Tear With a Continuous Sewing Machine–Like Suture Technique

**Fig 6:**
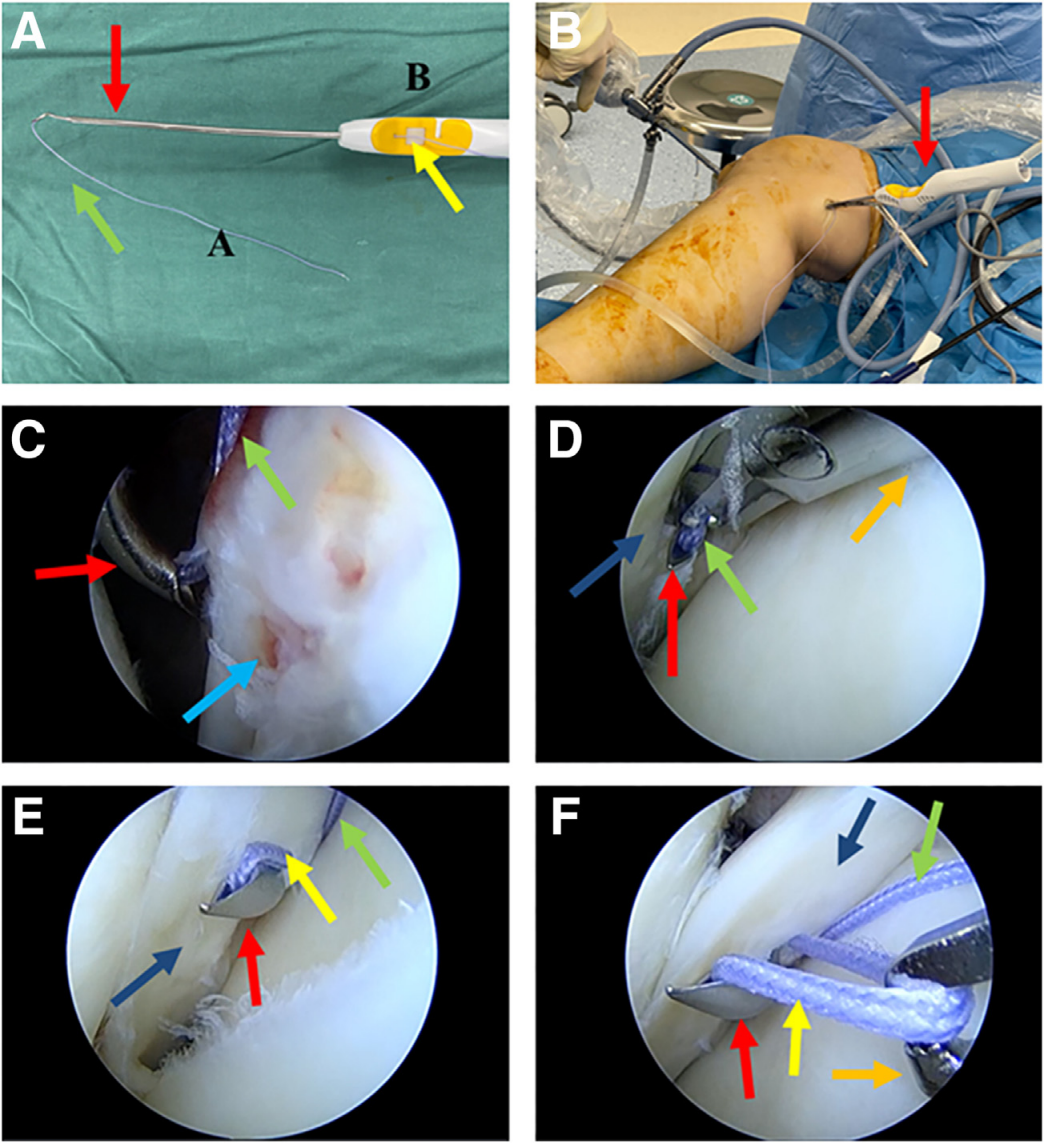
Through the posteromedial-transseptal portal, the continuous sewing machine–like suture technique is performed. Taking the first set of sutures as an example, each set of sutures includes 2 punctures on either side of the tear site. In actual application, multiple sets of sutures can be performed based on the shape and size of the tear. (A) Suture hook with sutures. The green arrow indicates the A end of the suture, the yellow arrow indicates the B end of the suture, and the red arrow indicates the suture hook. (B) The patient's position and portals used during suture process. (C) Using the anterolateral portal as the observation portal. The suture hook passes through the posteromedial-transseptal portal along with the retractor. During the first puncture of the first set of sutures, the suture hook enters close to the synovial edge of the lateral meniscus on the femoral side. (D) Using the anterolateral portal as the observation portal, during the first puncture of the first set of sutures, the suture hook exits from the tibial surface near the synovial edge of the lateral meniscus. The surgeon uses a grasper through the anteromedial portal to lift the posterior horn of the lateral meniscus to assist in exposing the tibial surface of the meniscus and pulls out the A end of the suture through the anteromedial portal. (E) Using the anterolateral portal as the observation portal, the suture hook performs the second puncture of the first set of sutures, and the suture hook exits from the tibial surface near the free edge of the lateral meniscus. (F) Using the anterolateral portal as the observation portal, a grasper enters through the anteromedial portal and pulls out the B end of the suture hook. The blue arrow indicates the femoral side of the torn lateral meniscus posterior horn, the dark blue arrow indicates the tibial surface of the torn lateral meniscus posterior horn, and the orange arrow indicates the grasper.

**Fig 7:**
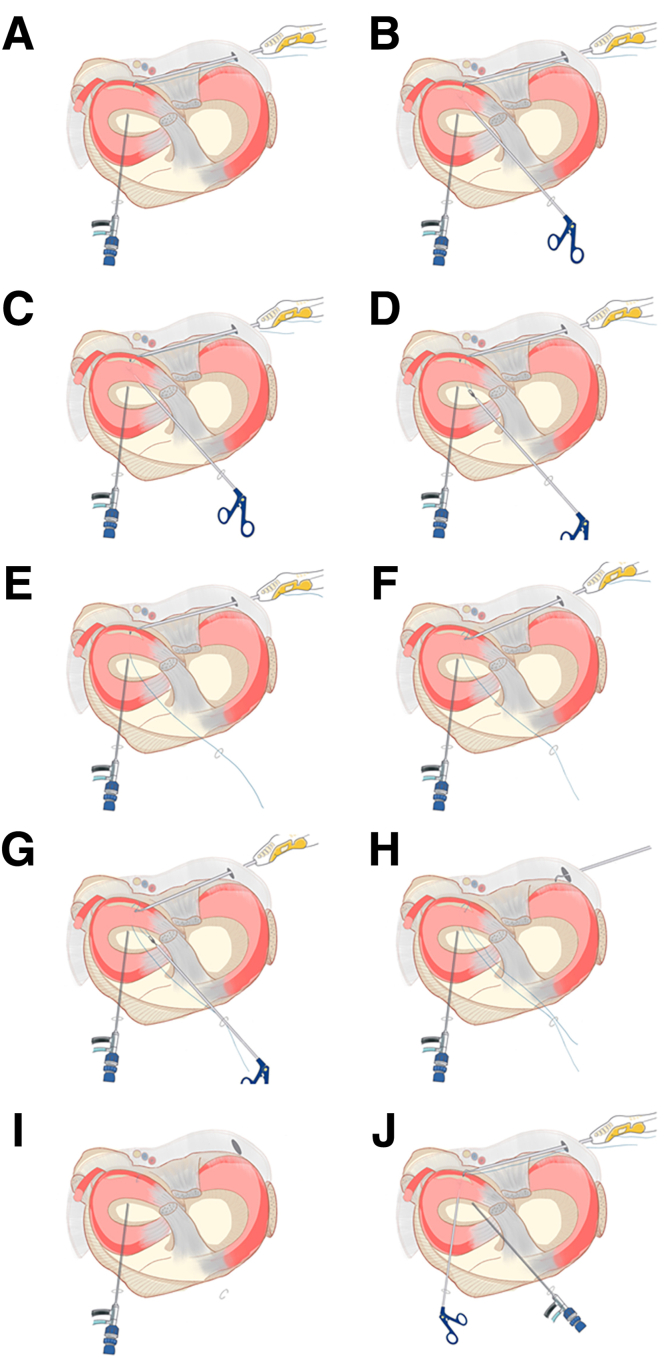
Schematic diagram of the first set of sutures performed through the posteromedial-transseptal portal. (A-E) The process of taking out the retractor and performing the first puncture of the first set of sutures. As shown in the figure, the suture hook passes from the synovial edge near the tear of the lateral meniscus posterior horn, piercing from femoral side to tibia side. A grasper enters through the anteromedial portal, lifting the synovial edge of the lateral meniscus posterior horn on the tibial surface to assist in exposing the exit point of the suturing hook and pulling out the A end of the suture through the anteromedial portal. (F-I) The process of the second puncture of the first set of sutures. As shown in the figure, the suture hook exits from the synovial edge of the lateral meniscus and punctures from the free edge near the tear of the lateral meniscus posterior horn, piercing from femoral side to tibia side. A grasper enters through the anteromedial portal, lifting the lateral meniscus posterior horn to assist in exposing the exit point of the suturing hook. The B end of the suture is pulled out through the anteromedial portal, and a knot is tied using a knot pusher through the anteromedial portal, with the knot placed on the tibial surface of the meniscus. (J) During the suturing process, the portal of the arthroscopic lens and the portal of grasper can be swapped for more convenient operation.

**Fig 8:**
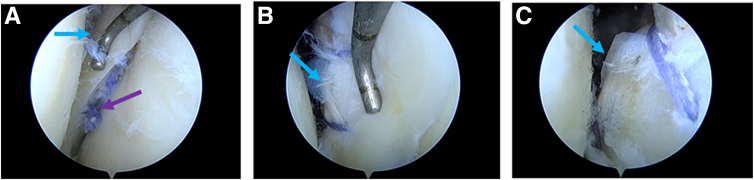
Arthroscopic views after meniscus suturing using our improved technique. (A) Using the anterolateral portal as the observation portal, the postsuturing view of the lateral meniscus tear site with sutures knotted on the tibial side of the meniscus is displayed. (B) Using the anterolateral portal as the observation portal, the view of the femoral side of the lateral meniscus tear site after suturing is shown. (C) Using the anteromedial portal as the observation portal, the view of the femoral side of the lateral meniscus tear site after suturing is presented. The blue arrow indicates the postsuturing lateral meniscus posterior horn, and the purple arrow indicates the suture knots placed on the tibial side of the meniscus.

**Fig 9:**
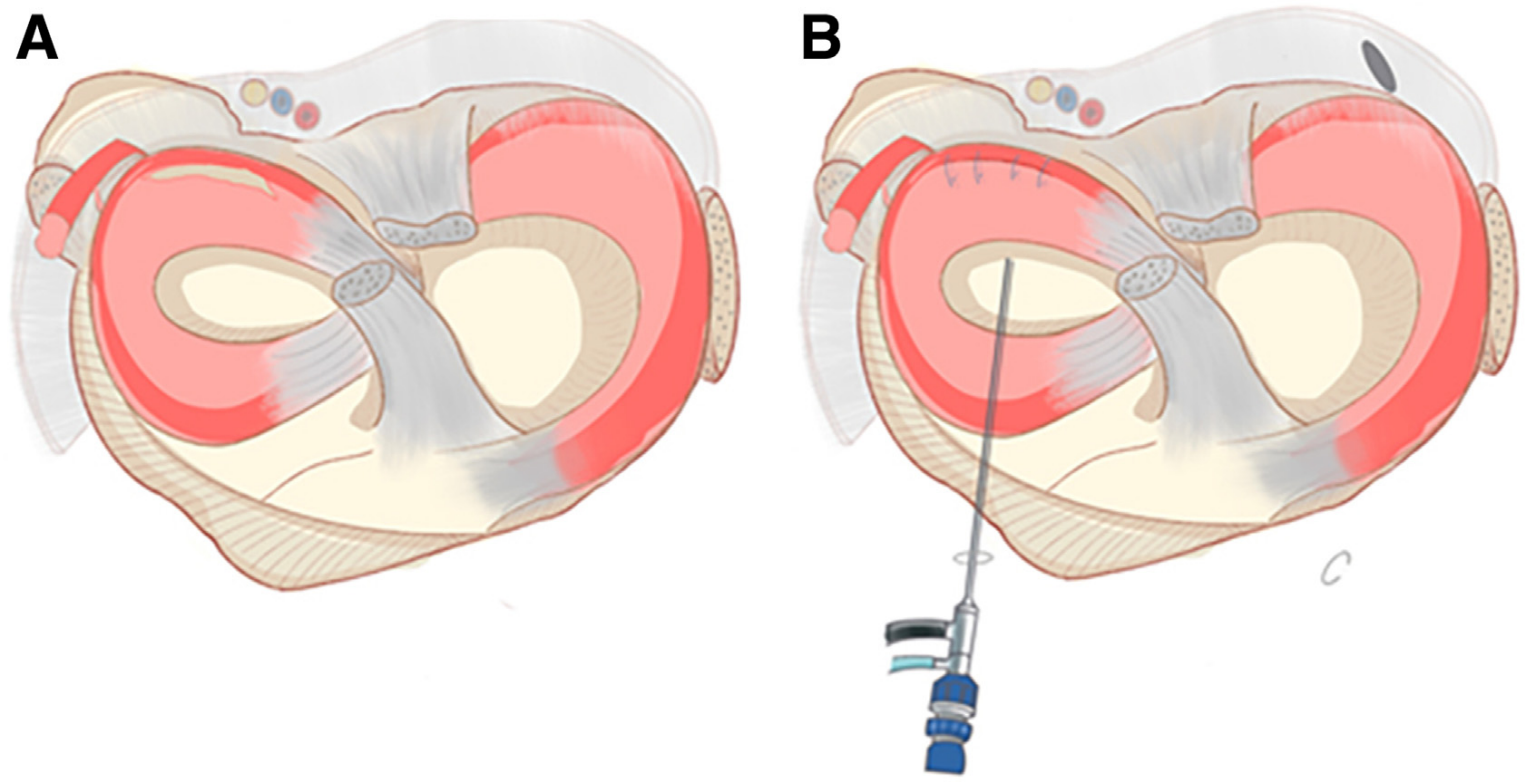
A schematic diagram of the postsuturing effect (here, 4 sets of postsuturing results are displayed). The comparison between the before and after suturing effects is demonstrated.

The surgeon can then begin the suture procedure. It should be pointed out that, according to previous reports,^2^ in our improved technique, the suture needs to leave an additional sufficient length (A end) at the head end of the suture hook (IDEAL Suture Shuttle, 45 Degrees Left; DePuy Mitek) (Fig 6A). The suture hook loaded with high-strength suture (Orthocord Suture; #2 Violet W/ MO-7 1/2 Circle, Taper Point Needle, 22 mm; DePuy Mitek) inserted into the posteromedial-transseptal portal is shown in Figure 6B, with intra-articular imaging presented in Figure 6C. With the suture hook inserted, the retractor can be withdrawn during the suture process. The operator can be free to choose the puncture point of the suture hook within the joint without fear of damaging the important vascular nerve tendon in the posterior-lateral area. Each suture can be punctured 2 times or more as needed. Here, we use the vertical mattress suturing process as an example to describe the suturing process of our improved technique (completed with 2 consecutive punctures) (Fig 6 C-E). The first puncture is made at the synovial edge of the tear. Then, the A end is pulled out through the anteromedial portal using a suture grasper. Subsequently, the suture hook exits from the meniscus. The second puncture is made at the free edge corresponding to the tear, and then the B end is pulled out through the anteromedial portal using a suture grasper. Finally, the A end and B end are knotted inside the joint through the anteromedial portal using a knot pusher. Each puncture is made by entering from the femoral side of the meniscus and exiting from the tibial side of the meniscus. The grasper can be used to lift the outer edge of the posterior horn of the lateral meniscus to assist in exposing the suture hook puncture site. It should be noted that the suture at the B end is holded by the operator until the suture is completed. When the last puncture in the suture process is completed, the B end suture is pulled out with grasper (Fig 6F). A set of schematic diagrams illustrating the suturing process, including 2 consecutive punctures, is shown in Figure 7A-I. It should be noted that anterolateral and anterolateral portals can be flexibly used as observation and operation portals (Fig 7J). In this example, due to the relatively regular longitudinal tear shape, we used continuous 2-puncture suturing, completing a total of 2 sets (4 punctures) of vertical mattress sutures. The meniscus morphology after suturing is shown in Figure 8. Here, the surgeon can place the suture knots on the tibial side surface of the meniscus rather than the femoral side surface, which will have a lesser impact on joint mobility. A diagram of the effect after suturing is shown in Figure 9 (we only showed 1 set of suturing in the previous figures). In the schematic diagrams presented here, we illustrate the results of 4 sets of vertical mattress sutures, totaling 8 punctures. It is worth noting that in the examples presented in this example, each set of sutures included only 2 consecutive punctures. According to our previous reports, more punctures are allowed; for example, an avulsion of the lateral meniscus posterior root may involve continuous 3- to 4-puncture sutures.^2^ The specific suture method should be determined by the surgeon based on the tear pattern.

## Discussion

Tearing of the lateral meniscus is a common sports medicine injury. The form of meniscal tears may vary in form and may often be combined with anterior cruciate ligament tears.^4^^,^^5^ Severe and complex lateral meniscus injuries have a significant impact on knee stability and usually require surgical treatment.^6^ The tear in the popliteal hiatus area of the lateral meniscus and the tear of posterior horn of the lateral meniscus are more challenging types in meniscus repair.^3^^,^^7^ Traditional suturing techniques through the anteromedial and anterolateral portals are relatively difficult to perform. In particular, the posterolateral part of the injury site is close to the popliteal tendon, popliteal blood vessel, and popliteal nerve, and a slight improper operation may cause serious complications. The use of an all-inside suture device is relatively simple, though. However, patients often have to bear a significant financial burden. Moreover, the lateral joint capsule is relatively thin, and fixation components are prone to dislodgement, leading to suture failure. Furthermore, compared to the standard vertical mattress sutures, all-inside suturing with a all-inside suture device is not a truly anatomic suture. Therefore, designing a safe, convenient, and cost-effective suturing method for the treatment of injuries in the popliteal hiatus area and posterior horn of the lateral meniscus is crucial.

In this report, we have mentioned an improved CUSP, which is based on our previous research on the enhancement of meniscus suturing techniques and surgical portals.^2^^,^^3^ In this technique, we have made further updates based on the previous reported technique.^2^^,^^3^ The innovations primarily lie in the following 3 aspects:

1. In the process of establishing the posteromedial-transseptal portal, we enter the arthroscope into the posteromedial compartment through the gap between the posterior cruciate ligament and the medial condyle of the femur, rather than the intercondylar fossa portal. This is a significant innovation. This further reduces the possibility of cruciate ligament injury, resulting in reduced trauma and simplified surgical procedures. It is important to note that during this process, the knee joint’s position needs to be changed from flexed at 15 to 30 degrees to the “figure-4” position. In our previous reports,^3^ it has been mentioned that the establishment of the posteromedial-transseptal portal is generally safe, as there are no important blood vessels, nerves, or tendons within the posterior midline compartment of the knee joint. When the suture hook or other operating tools such as probe pass through this portal, they can directly reach the injury site. At this point, the operating angle of tools like the suture hook and the field of view of the arthroscope are both optimal, significantly reducing the exposure difficulty and operational challenges for the surgeon. Furthermore, after passing through this portal, the suture hook can more easily reach the damaged area, whether it is near the synovial edge or the popliteomeniscal fascicles, giving it an advantage over traditional suturing techniques.
2. During the insertion, the suture hook is likely to get stuck in the soft tissue, and there is a risk of damaging blood vessels and nerve tissues when the insertion direction is incorrect. Therefore, we have made another improvement in this technique, that is, the introduction of retractor protection technology, which makes the establishment and operation of this improved portal safer and faster, so that interference by soft tissue with the suture hook is reduced. Additionally, the use of a retractor can prevent damage to the popliteal vessels and nerves caused by the suture hook, enhancing the safety of the surgery. Due to the protective role of the retractor and its ability to isolate soft tissue, the suture hook enters smoothly through the portal without needing whole-process arthroscopic monitoring and tissue cleaning with plasma radiofrequency. The retractor can be withdrawn during the suturing procedure, thereby increasing the range of motion for the suture hook. Therefore, the retractor is highly maneuverable and flexible.
3. In our previous reports,^3^ a “suture loop” suturing technique was used for longitudinal meniscus tears. It should be noted that this “suture loop” technique is not the most ideal or preferred method, and the best suturing approach for the tear area should be the standard vertical mattress suture. To address this issue and improve suturing efficiency, we have introduced the previously reported “continuous sewing machine–like suture technique” for suturing posterior lateral meniscal injuries.^2^ In this instance, for the longitudinal tear of the posterior lateral meniscus, we employed this combined technique. Surgeons can use this improved continuous suturing method to achieve the classic vertical mattress sutures, which result in the best healing of the meniscus with minimal interference. Furthermore, after the suturing is completed, the surgeon can tie the suture knots on the tibial side of the meniscus. In traditional suture hook suturing methods, surgeons typically tie the suture knots on the femoral side of the meniscus, which can to some extent affect joint mobility and lead to unnecessary wear on the joint surface. In addition, the tibial side of the meniscus where the suture knot is left is suitable for additional transtibial pullout repair. Our improved technique is particularly suitable for posterior root injuries of the lateral meniscus. With our technique, clinicians can perform multiple consecutive sutures to achieve the fixation of the meniscus root. As the suture knot itself is located at the tibial end, direct pulling out through the tibial tunnel is highly appropriate. Surgeons can flexibly choose the puncture location of the suture hook based on the type of injury, making it highly applicable for various complex lateral meniscus posterior horn/body tear patterns. For instance, when dealing with different types of tears in the popliteal hiatus area of the lateral meniscus and the posterior root of the lateral meniscus, this portal and the continuous suturing method significantly increase the speed of suturing, making it simpler and quicker. More important, the devices used in the CUSP we reported are commonly used in meniscus suturing, without the need for high-value consumables. This enhances the cost-effectiveness and feasibility of this surgical technique. The pearls and pitfalls of our improved technique are shown in Table 1, while the advantages and disadvantages are shown in Table 2. What needs to be pointed out here is that, although the posteromedial portal needs to be established, in many cases, however, it is necessary to create the posteromedial portal to address simultaneous medial meniscus injuries such as RAMP injury or posterior horn tear. The posteromedial portal is a commonly established portal, so it does not increase the risk of our designed modified technique, nor does it affect the simplicity and minimally invasive characteristics of our modified technique. Objectively speaking, our improved technique integrates economy, simplicity, and safety and has good clinical application value.

**Table 1:** The Pearls and Pitfalls of the Improved Chinese Union Suture Procedure

Pearls	Pitfalls
1. When establishing the posteromedial portal, the arthroscope can directly enter the posteromedial compartment through the gap between the posterior cruciate ligament and the medial condyle of the femur, avoiding damage to surrounding tissues that can occur with the intercondylar fossa portal. 2. When establishing the posteromedial portal, the blunt trocar obturator is inserted and the posterior mediastinum is bluntly traversed directly from behind the posterior cruciate ligament to the posterior external compartment under arthroscopic supervision.	1. When using a blunt trocar obturator for blunt puncture from the posteromedial compartment to the posterolateral compartment, it is important to control the direction carefully to avoid heading toward the location of the popliteal neurovascular bundle. 2. When suturing around the popliteal hiatus area of the lateral meniscus, the suture hook should be used under arthroscopic monitoring to avoid damaging the popliteal tendon.
3. The retractor can effectively protect the posterior neurovascular bundle in the popliteal fossa when the suture hook enters through the posteromedial-transseptal portal. Retractor also makes it easier for equipment to enter.	
4. The front end of the suture hook should enter the joint in close proximity to the retractor to prevent it from getting caught in soft tissues or causing damage to soft tissues.	
5. Once the suture hook enters the posterolateral compartment of the knee joint, the retractor can be pulled out, allowing for increased mobility of the suture hook within the joint.	
6. During the suturing process, the suture hook can be operated without being pulled out of the joint, as long as the suture is long enough.	
7. The anteromedial portal and anterolateral portal can be switched freely between the observation portal and the operation portal, making both observation and operation more efficient.	
8. Tightening both ends of the suture (A and B ends) on the suture hook can help prevent damage to the suture thread.

**Table 2:** The Advantages and Disadvantages of the Improved Chinese Union Suture Procedure

Advantages	Disadvantages
1. This technique has a technical advantage for tears of the lateral meniscus that are close to the synovial edge. The suture hook can easily reach the edge tissues, and continuous suturing allows for the completion of classic vertical mattress sutures, resulting in the best healing of the meniscus with minimal interference.	1. In this technique, the operator needs to create a posteromedial portal, which may cause additional tissue damage. 2. There is a risk of damage to the popliteal vascular and nerve during the establishment of the portal and the process of suture hook to enter and exit the joint.
2. This technique can address various complex tears of the lateral meniscus body and posterior horn, including posterior root, making it versatile in application.	
3. This technology improves upon the original, with blunt tools such as the blunt trocar obturator and retractor to further improve the safety of approach creation and suture hook operation.	
4. The devices required for this technique are based on commonly used meniscus suturing devices, and they do not require high-cost consumables. This makes the technique cost-effective and highly suitable for wider adoption.	
5. This technique combines a modified portal with a modified suturing method, maximizing the advantages of both and further simplifying the suturing of lateral meniscus injuries.	
6. In this technique, the suture knots can be tied on the tibial side of the meniscus, resulting in less impact on joint mobility and the femoral articular surface. This technique is especially suitable for transtibial pullout repair.

## Supplementary Data

supplementary material

Video 1 Introduction of improved Chinese Union Suture Procedure (CUSP). The patient has a right knee injury. The surgeon first positions the knee joint at 15-30 degrees of flexion and uses the anterolateral portal as the observation portal, inserting the arthroscope into the posterior medial compartment to prepare for guiding the establishment of the posteromedial portal. Then, the knee joint is positioned in the "Figure-4 sign," establishing a standard posterior medial approach, and the trocar core is inserted through the posterior medial approach into the posterior medial compartment. The specific suture method is continuous sewing machine-like suture technique.
